# Esculin protects against methionine choline-deficient diet-induced non-alcoholic steatohepatitis by regulating the Sirt1/NF-κB p65 pathway

**DOI:** 10.1080/13880209.2021.1945112

**Published:** 2021-07-09

**Authors:** Xi-Ding Yang, Zhuo Chen, Ling Ye, Jing Chen, Yong-Yu Yang

**Affiliations:** a Department of Pharmacy, The Second Xiangya Hospital of Central South University, Changsha, Hunan, China; b Hunan Provincial Engineering Research Central of Translational Medical and Innovative Drug, The Second Xiangya Hospital of Central South University, Changsha, Hunan, China; c Department of Geriatrics, The Third Xiangya Hospital of Central South University, Changsha, Hunan, China; d Department of Geriatrics, The Second Xiangya Hospital of Central South University, Changsha, Hunan, China; e Department of Pharmacology, Xiangya School of Pharmaceutical Sciences, Central South University, Changsha, Hunan, China

**Keywords:** Inflammatory cytokine, acetylation of NF-κB p65, fibrosis

## Abstract

**Context:**

Esculin, an active coumarin compound, has been demonstrated to exert anti-inflammatory effects. However, its potential role in non-alcoholic steatohepatitis (NASH) remains unclear.

**Objective:**

This study explored the hepatoprotective effect and the molecular mechanism of esculin in methionine choline-deficient (MCD) diet-induced NASH.

**Materials and methods:**

Fifty C57BL/6J mice were divided into five groups: control, model, low dosage esculin (oral, 20 mg/kg), high dosage esculin (oral, 40 mg/kg), and silybin (oral, 105 mg/kg). All animals were fed a MCD diet, except those in the control group (control diet), for 6 weeks.

**Results:**

Esculin (20 and 40 mg/kg) inhibited MCD diet-induced hepatic lipid content (triglyceride: 16.95 ± 0.67 and 14.85 ± 0.78 vs. 21.21 ± 1.13 mg/g; total cholesterol: 5.10 ± 0.34 and 4.08 ± 0.47 vs. 7.31 ± 0.58 mg/g), fibrosis, and inflammation (ALT: 379.61 ± 40.30 and 312.72 ± 21.45 vs. 559.51 ± 37.01 U/L; AST: 428.22 ± 34.29 and 328.23 ± 23.21 vs. 579.36 ± 31.93 U/L). *In vitro*, esculin reduced tumour necrosis factor-α, interleukin-6, fibronectin, and collagen 4A1 levels, but had no effect on lipid levels in HepG2 cells induced by free fatty acid. Esculin increased Sirt1 expression levels and decreased NF-κB acetylation levels *in vivo* and *in vitro*. Interfering with Sirt1 expression attenuated the beneficial effect of esculin on inflammatory and fibrotic factor production in HepG2 cells.

**Conclusions:**

These findings demonstrate that esculin ameliorates MCD diet-induced NASH by regulating the Sirt1/ac-NF-κB signalling pathway. Esculin could thus be employed as a therapy for NASH.

## Introduction

Non-alcoholic fatty liver disease (NAFLD) is currently recognized as the most common chronic liver disease globally and is an important cause of the increased risk of end-stage liver disease, hepatocellular carcinoma, and liver-related mortality (Isaza et al. 2020). NAFLD has various pathological spectra, including simple fatty liver, non-alcoholic steatohepatitis (NASH), liver fibrosis, and cirrhosis (Gluchowski et al. 2017). NASH, characterized by liver steatosis, hepatocellular injury, inflammation, and different degrees of fibrosis, is a key progression from simple hepatosteatosis to liver fibrosis (Gonzalez et al. 2020). Therefore, timely and effective treatment of NASH is required to prevent further development into liver fibrosis, cirrhosis, and even hepatocellular carcinoma (De Lorenzo et al. 2020). Currently, there are no effective drugs approved for the treatment of NASH (Meng et al. 2019), which interferes with the lifestyle of patients. As hepatoprotective drugs are commonly used in the clinic to improve the symptoms of patients with NASH (Noureddin et al. 2020), discovering safe and effective drugs to treat NASH is urgently warranted.

The pathogenesis of NASH involves a complex mechanism, including insulin resistance, lipotoxicity, oxidative stress, inflammation, and cell apoptosis (Kuchay et al. 2020). Among them, inflammation plays a central role in the development of NASH (Kragh et al. 2020). NF-κB p65 is a pivotal transcription factor that regulates the expression of inflammatory signalling molecules (Gasparini and Feldmann 2012). Based on previous studies, the acetylation of RelA (ac-NF-κB), an important post-translational modification process, has an important role in the retention of NF-κB activity in the nucleus (Huang et al. 2010). Sirtuin type 1 (Sirt1) is a histone deacetylase that is dependent on nicotinamide adenine dinucleotide (NAD^+^) and is involved in cell proliferation, differentiation, senescence, apoptosis, and metabolism (Pardo and Boriek 2020). Sirt1 deacetylates the lysine residue of NF-κB (RelA/p65) at position 310 to inhibit the transcriptional activity of NF-κB and reduce the expression of proinflammatory cytokines (Yeung et al. 2004). Therefore, improving Sirt1/ac-NF-κB signalling might be beneficial for NASH.

**Figure 1.**
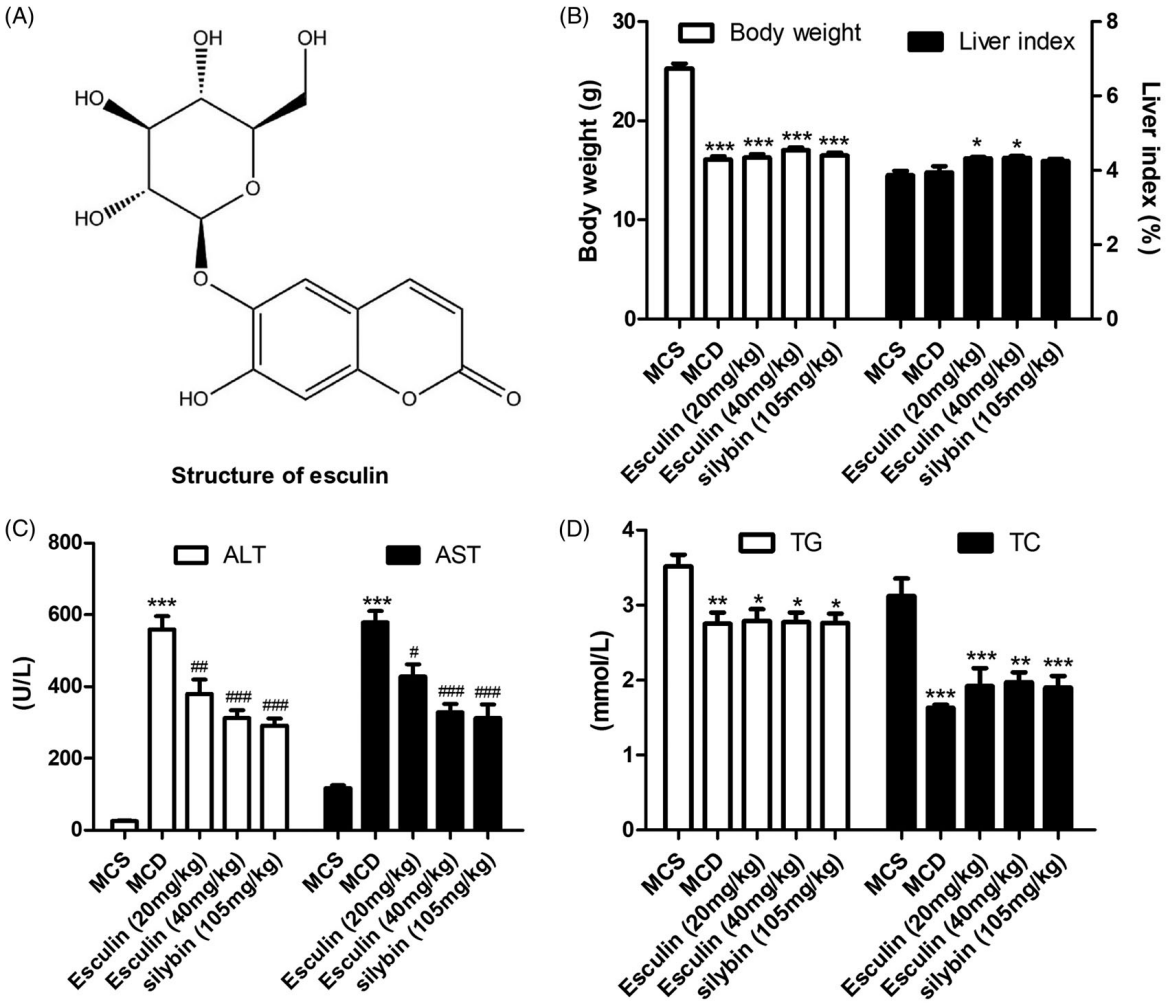
Esculin attenuates MCD diet-induced ALT and AST levels in serum. (A) Structure of esculin. (B) Body weight and liver index. (C) Level of ALT and AST in serum. (D) Level of TC and TG in serum. Data are expressed as mean ± SEM, *n* = 3. *p < 0.05, **p < 0.01, ***p < 0.001 *vs.* MCS group. ^#^*p* < 0.05, ^##^*p* < 0.01, ^###^*p* < 0.001 *vs.* MCD group. MCS: methionine and choline supplemented; MCD: methionine and choline-deficient.

Esculin (Figure 1(A)), a major active component of the stem bark of *Fraxinus rhynchophylla* Hance (Oleaceae), exerts multiple pharmacological effects, including antibacterial (Mokdad-Bzeouich et al. 2015), antioxidant (Biljali et al. 2012), anti-inflammatory (Niu et al. 2015), antitumor (Mokdad-Bzeouich et al. 2016), and anti-diabetic nephropathy (Kang et al. 2014). Previous studies have demonstrated that esculin plays a protective role in lipopolysaccharide (LPS)/d-galactosamine-induced acute liver injury (Liu et al. 2018). Importantly, this hepatoprotective effect is associated with its inhibition of the NF-κB p65 signalling pathway (Liu et al. 2018). Esculin has also been shown to inhibit the levels of tumour necrosis factor-α (TNF-α) and interleukin-6 (IL-6) in LPS-stimulated mouse peritoneal macrophages (Niu et al. 2015) and LPS-induced acute lung injury in mice by regulating the NF-κB pathway (Tianzhu and Shumin 2015). Therefore, we speculate that esculin may have a protective effect against NASH.

In the present study, we demonstrate that esculin exhibits hepatoprotective effects against MCD diet-induced NASH. Mechanistically, the Sirt1/ac-NF-κB p65 signalling pathways were found to be regulated by esculin.

## Materials and methods

### Materials

Esculin (purity ≥98%, lot # D1812002, purchased year: 2019) and oleic acid (lot # I1608084) were purchased from Aladding Company Ltd. (Shanghai, China). Oil Red O was (purity ≥75%, lot # 303C051, purchased year: 2018) purchased from Solarbio Science and Technology Company (Beijing, China). Lipofectamine 3000 Transfection Reagent was purchased from Invitrogen (Carlsbad, CA, USA). The anti-NF-κB p65 acetyl antibody was obtained from Abcam (Cambridge, MA, USA). The anti-fibronectin, anti-collagen 4A1, and anti-β-actin antibodies, and the TNF-α, IL-6, and IL-1β ELISA kits were purchased from Boster Biological Technology, Ltd. (Wuhan, China). Triglyceride (TG), total cholesterol (TC), and free fatty acid (FFA) detection kits were procured from Nanjing Jiancheng Bioengineering Institute (Nanjing, China). Anti-Sirt1 antibody, anti-NF-κB p65 antibody, the bicinchoninic acid (BCA) protein assay kit, radioimmunoprecipitation assay (RIPA), phenylmethylsulfonyl fluoride (PMSF), NADPH/NADP^+^ ratio, and the BeyoECL plus kit were purchased from Beyotime Biotechnology (Shanghai China). TRIzol, SYBR^®^ Premix Ex Taq™, and the PrimeScript reverse transcription reagent kit were purchased from Takara Biotechnology Co., Ltd. (Dalian, China). Methionine-choline deficient (MCD) diet and methionine and choline supplemented (MCS) diet (control diet) were purchased from Shulaibao Biotechnology Co., Ltd. (Wuhan, China). Dulbecco’s modified Eagle’s medium (DMEM) and foetal bovine serum (FBS) were obtained from HyClone (Logan, UT, USA). FFA-free bovine serum albumin (BSA, lot # D00162664) and palmitic acid (lot # SLBG3957V) were procured from Sigma-Aldrich (St Louis, MO, USA).

### Animals and experimental protocol

All animal studies were approved by the Animal Ethical and Welfare Committee of the Second Xiangya Hospital of Central South University (No. 2020254) and followed the guidelines of the National Institutes of Health. Male C56BL/6J mice (age, 8 weeks old; weight, 20 ± 2 g) were purchased from SiLaiKeJingDa Laboratory Animal Ltd. (Changsha, China), and housed in a temperature-controlled room (24 ± 1 °C) under a 12 h light/dark cycle. A total of 50 mice were randomly divided into the following five groups (*n* = 10): Control group, model group, low-dosage esculin group (20 mg/kg), high-dosage esculin group (40 mg/kg) (Liu et al. 2018), and positive drug group (silybin, 105 mg/kg) (Ou et al. 2018). Mice in the model group, the esculin-treated groups, and the positive drug groups were fed an MCD diet for 6 weeks; and mice in the normal control group were fed an MCS diet. The composition of the diets was shown in Table 1. Mice in the esculin-treated groups were intragastrically administered different dosages of esculin (dissolved in PBS) daily during diet feeding, and mice in the control and model groups were intragastrically administered equal volumes of PBS. After 6 weeks of treatment, mice were anaesthetized with 2% sodium pentobarbital, and blood was collected from the retro-orbital sinus after eyeball removal. Serum samples were obtained from whole blood via centrifugation at 4 °C for 10 min, and liver samples were collected for histological and molecular examination.

**Table 1.** Composition of the MCS diet and MCD diet formulas.

Ingredients (g)	MCS diet	MCD diet
l-cystine	4.2	4.2
l-isoleucine	7.6	7.6
l-leucine	15.8	15.8
l-lysine	13.2	13.2
l-methionine	5.1	0.8
l-phenylalanine	8.4	8.4
l-threonine	7.2	7.2
l-tryptophan	2.1	2.1
l-valine	9.3	9.3
l-histidine	4.6	4.6
l-alanine	5.1	5.1
l-arginine	6.0	6.0
l-aspartic acid	12.1	12.1
l-glutamic acid	38.2	38.2
glycine	3.0	3.0
l-proline	17.8	17.8
l-serine	10.0	10.0
l-tyrosine	9.2	9.2
corn starch	315	319.3
maltodextrin 10	35	35
sucrose	350	350
cellulose	50	50
soybean oil	25	25
lard	20	20
mineral mix S10026	10	10
dicalcium phosphate	13	13
calcium carbonate	5.5	5.5
potassium citrate	16.5	16.5
sodium bicarbonate	7.5	7.5
vitamin mix V10001	10	10
choline	2	0

### Cell culture and cell treatment

HepG2 cells were purchased from American Type Culture Collection (ATCC, Manassas, VA, USA) and cultured in DMEM supplemented with 10% FBS in a humidified atmosphere of 5% CO_2_ at 37 °C. The FFA (oleic acid: palmitic acid = 2:1) was dissolved in 10% fat-free BSA. When cell confluence reached 50%, the cells were incubated with FFA (500 µM) in the presence and absence of esculin for 24 h. Cells in the control group were treated with 1% BSA only.

### Cellular viability analysis

The effect of esculin on the viability of HepG2 cells was measured using the MTT method. Briefly, HepG2 cells were seeded in a 96-well plate. After reaching 50% confluence, the cells were treated with and without different concentrations of esculin (12.5, 25, 50, 100, and 200 µM) for 24 h. Subsequently, the cells were incubated with MTT for 4 h at 37 °C, and the formed formazan was dissolved with 200 µL dimethylsulphoxide. The optical density value was measured at 450 nm using an Infinite F50 microplate reader (Tecan, Mannedorf, Switzerland). The results are expressed as the relative percentage of the absorbance value of esculin-treated cells compared to that of control cells. The experiment was performed in triplicate.

### Histology staining and immunohistochemistry analysis

The liver tissue was fixed in 4% paraformaldehyde and embedded in paraffin. Paraffin blocks were cut into 5 µm-thick sections for haematoxylin and eosin (H&E) and Masson’ trichrome staining. For immunohistochemical staining, the sections were incubated with anti-F4/80, collagen 4A1, or fibronectin antibodies overnight at 4 °C, respectively. The sections were then incubated with biotinylated secondary antibodies for 1 h at room temperature. DAB reagent was used to detect positive staining, and the sections were counterstained with haematoxylin. Finally, images were captured with an Olympus IX73 microscope (Tokyo, Japan).

### Oil red O staining analysis

The liver tissue was fixed in 10% paraformaldehyde and stored in a −80 °C freezer. All tissues were cryo-sectioned into 10 µm-thick sections and incubated with the Oil Red O working solution for 15 min at room temperature. The sections were washed with deionized water and subjected to haematoxylin staining. Finally, the sections were sealed with glycerine gelatine and observed with an Olympus IX73 microscope (Tokyo, Japan). Oil red O staining was also used to determine the level of lipid accumulation in HepG2 cells. After treatment, HepG2 cells were washed three times with PBS and then fixed with 4% paraformaldehyde for 30 min. Thereafter, the cells were washed with 60% isopropanol and incubated with Oil Red O working solution for 30 min at room temperature. Subsequently, the HepG2 cells were washed three times with 60% isopropanol, and isopropanol was added to extract the Oil Red O dye. The optical density (OD) value was determined using a microplate reader at a wavelength of 520 nm.

### Western blot analysis

Liver tissue and the collected cells were lysed using RIPA buffer supplemented with PMSF for 30 min, and centrifuged at 12,000 *g* at 4 °C for 10 min. The supernatant was collected, and the protein concentration was determined using the BCA kit. Protein (35 µg) was added to each lane and separated by 10% SDS polyacrylamide gel electrophoresis. The proteins were then transferred to polyvinylidene difluoride membranes, which were subsequently blocked with 5% non-fat milk for 1 h. The membranes were incubated with primary antibody at 4 °C overnight, followed by anti-rabbit or anti-mouse secondary antibodies for 1 h. The bolt was detected with ECL reagent and captured using an Amersham Imager 600 (Little Chalfont, UK). Density analysis of the blot was performed using Image J 1.37c software (National Institute of Health).

### Evaluation of TG, TC, and FFA in the liver tissue

A total of 100 mg of liver tissue from mice in each group were homogenized on ice using PBS. The lysis solution was centrifuged at 12,000 *g* for 15 min at 4 °C. The supernatants were collected and used to determine the levels of TG, TC, and FFA using the commercial standard enzymatic assay kits.

### Evaluation of AST, ALT, TG, and TC levels

The levels of alanine aminotransferase (ALT), aspartate aminotransferase (AST), TG and TC in serum, and the levels of ALT and AST in the cell culture supernatant were determined using a biochemical analyzer.

### siRNA analysis

Sirt1 siRNA and scrambled siRNA were purchased from RiboBio (Guangzhou, China). Transfection was carried out according to the manufacturer’s instructions. Briefly, siRNA and scrambled RNA were diluted with serum-free DMEM. Thereafter, lipofectamine 3000 transfection was added and incubated for 15 min. The mixed solution was added to a culture plate and incubated with cells at 37 °C, 5% CO_2_ for 24 h. Following transfection, a Western blot was performed to verify the efficiency of gene silencing.

### Quantitative real-time PCR

Total RNA was extracted from the liver tissue or HepG2 cells using TRIzol reagent. Thereafter, a reverse transcription reaction was carried out using the PrimeScript reverse transcription reagent kit. The conditions for the reverse transcription reaction were 42 °C for 15 min, 85 °C for 5 s, and maintenance at 4 °C. Quantitative PCR (qPCR) was performed to measure gene expression using a LightCycler96^®^ instrument (Roche Diagnostics GmbH) and the SYBR^®^ Premix Ex Taq™ reagent. The following species-specific primers were employed: Mouse ADRP forward primer: 5′-CAAGCACAGCGACGAGTACAAGATCC-3′ and reverse primer: 5′-GCTTGAACAAGTTCCGCAGGGTG-3′; mouse FASN forward primer: 5′-CCGTGTGACCGCCATCTATATCG-3′ and reverse primer: 5′-CGTGAGGTTGCTGTCGTCTGTAG-3′; mouse CD36 forward primer: 5′-CCCTAACCCAAAGGAGCATT-3′ and reverse prime: 5′-CACAGCAACGGCAGAACTAC-3′; mouse β-actin forward primer: 5′-CCAGGGTGTGATGGTGGGAATG-3′ and reverse primer: 5′-CGCACGATTTCCCTCTCAGCTG-3′; human TNF-α forward primer: 5′-AGCTGGTGGTGCCATCAGAGG-3′ and reverse prime: 5′-TGGTAGGAGACGGCGATGCG-3′; human IL-6 forward primer: 5′-CACTGGTCTTTTGGAGTTTGAG-3′ and reverse prime: 5′-GGACTTTTGTACTCATCTGCAC-3′; human β-actin forward primer: 5′-CTCTTCCAGCCTTCCTTCCT-3′ and reverse primer: 5′-AGCACTGTGTTGGCGTACAG-3′. The qPCR cycling conditions were 94 °C for 5 s, followed by 45 cycles of 95 °C for 5 s and 60 °C for 30 s. The results were calculated using the 2^−ΔΔCq^ method. All experiments were carried out in triplicate.

### Immunofluorescence analysis

The paraffin sections were deparaffinized, dehydrated, and subjected to antigen retrieval. Thereafter, 3% hydrogen peroxide was used to block the endogenous peroxidase activity. The sections were then incubated with 5% goat serum for 30 min and incubated with primary antibodies at 4 °C overnight, followed by incubation with a secondary antibody for 1 h at room temperature. Finally, the nuclei were stained with 4′,6-diamidino-2-phenylindole (DAPI) for 10 min. Images were captured with a fluorescence microscope (Zeiss AG, Oberkochen, Germany).

### Inflammatory factor analysis

The levels of TNF-α, IL-1β, and IL-6 in the liver tissue or cell culture supernatant were determined using ELISA kits, according to the manufacturer’s protocols.

### Statistical analysis

The experiment data are expressed as mean ± S.E.M. The data were analyzed using one-way analysis of variance (ANOVA) followed by Tukey’s test on GraphPad Prism 6 (GraphPad Software Inc., La Jolla, CA, USA). *p* < 0.05 was considered to indicate a statistically significant difference.

## Results

### Esculin attenuates the MCD diet-induced increase in ALT and AST levels in serum

The effect of esculin on MCD diet-induced NASH was assessed. Compared to mice in the control group (MCS group), the body weight of mice in the MCD group was significantly decreased while the liver index was markedly increased. Treatment with esculin was not found to alleviate these changes compared to MCD (Figure 1(B)). Further, mice fed an MCD diet had a significant increase in the levels of ALT and AST in serum; however, these levels were significantly reduced by esculin (20 and 40 µM) (Figure 1(C)). The effect of esculin on blood lipid in NASH mice was also evaluated. As shown in Figure 1(D), the levels of TC and TG in serum were significantly decreased following MCD treatment; this may be caused by a defect in the hepatic secretion of TG (Kim et al. 2017). However, treatment with esculin had no effect on the levels of TC and TG. Together, these findings suggest that esculin effectively inhibits the development of hepatic inflammation.

### Esculin attenuates MCD diet-induced hepatic lipid accumulation

**Figure 2.**
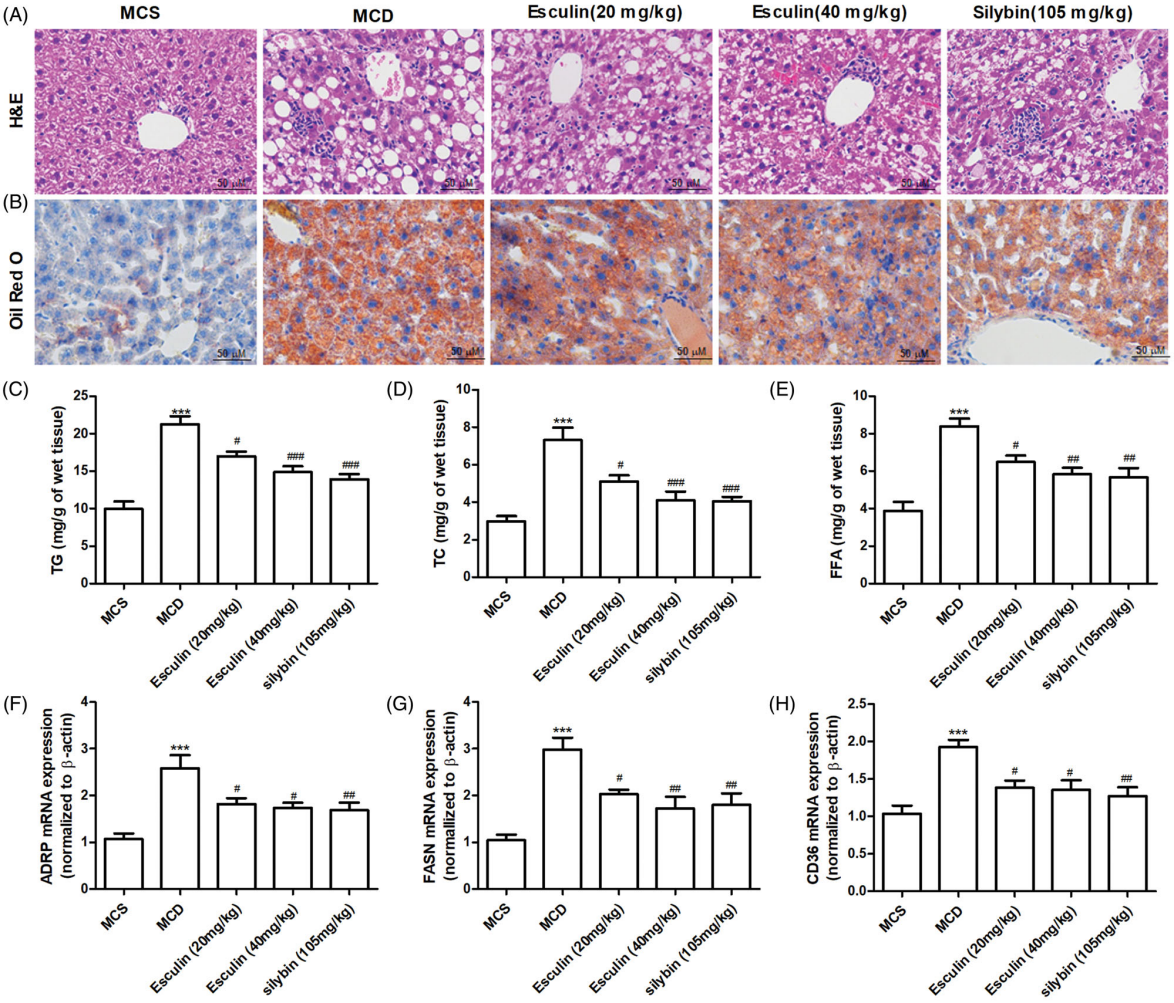
Esculin attenuates MCD diet-induced hepatic lipid accumulation. (A) Representative H&E staining images of the liver section from each group (400× magnification, scale bar: 50 µm). (B) Representative Oil Red O staining images of the liver section from each group (400× magnification, scale bar: 50 µm). (C) Level of TG in the liver tissue. (D) Level of TC in the liver tissue. (E) Level of FFA in the liver tissue. (F) Level of ADRP mRNA in the liver tissue. (G) Level of FASN mRNA in the liver tissue. (H) Level of CD36 mRNA in the liver tissue. Data are expressed as mean ± SEM, *n* = 3. ***p < 0.001 *vs.* MCS group. ^#^*p* < 0.05, ^##^*p* < 0.01, ^###^*p* < 0.001 *vs.* MCD group. MCS: methionine and choline supplemented; MCD: methionine and choline-deficient.

To investigate the effects of esculin on the changes in liver pathology in mice with MCD diet-induced NASH, H&E and Oil red O staining were performed. Based on H&E staining, macrovesicular steatosis was significantly increased in the mice group fed an MCD diet compared to mice in the MCS group. However, treatment with esculin was found to reduce the MCD diet-induced pathological changes (Figure 2(A)). The result of the Oil red O staining revealed that the level of lipid accumulation in the liver tissue was significantly increased in the MCD group compared to the MCS group, and esculin treatment could effectively attenuate the increased lipid accumulation (Figure 2(B)). We measured the content of lipid components, such as TG, TC, and FFA in the liver tissue. As shown in Figure 2(C–E), esculin prevented the MCD diet-induced increase in the levels of TG, TC, and FFA in the liver tissue. Similarly, we confirmed that the expression of the lipid synthesis genes, adipose differentiation-related protein (*ADRP*) and fatty acid synthase (*FASN*), and fatty acid translocase (FAT/CD36) were markedly upregulated in mice fed the MCD diet; however, this upregulation was ameliorated in esculin-treated mice (Figure 2(F–H)).

### Esculin attenuates MCD diet-induced fibrosis and inflammation

**Figure 3.**
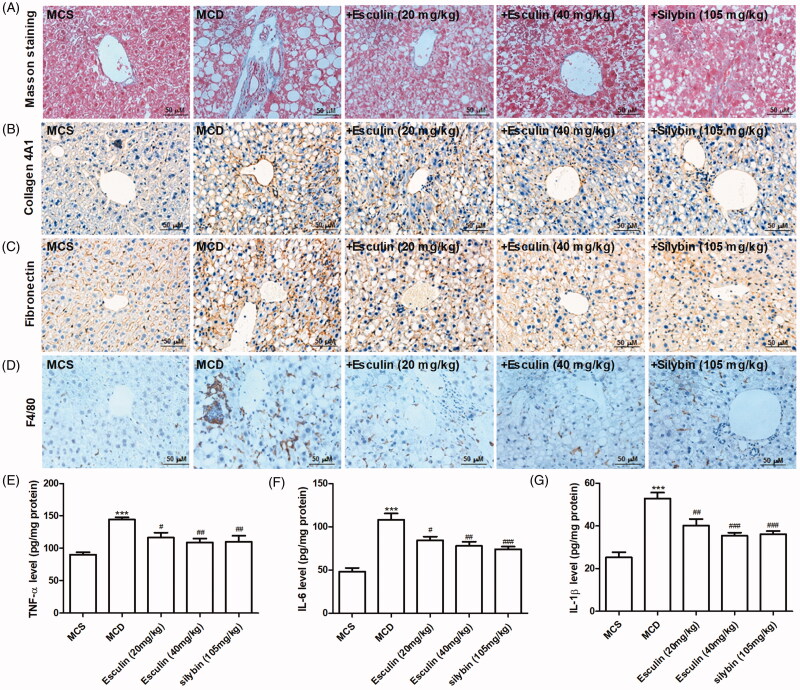
Esculin attenuates MCD diet-induced fibrosis and inflammation. (A) Representative Masson trichrome staining images of the liver section from each group (400× magnification, scale bar: 50 µm). (B) Representative images from the immunohistochemistry analysis of the collagen 4A1 protein in the liver of each group (400× magnification, scale bar: 50 µm). (C) Representative immunohistochemical images of the fibronectin protein in the liver of each group (400× magnification, scale bar: 50 µm). (D) Representative immunohistochemical images of the F4/80 protein in the liver of each group (400× magnification, scale bar: 50 µm). (E) Level of TNF-α in the liver tissue. (F) Level of IL-6 in the liver tissue. (G) Level of IL-1β in the liver tissue. Data are expressed as mean ± SEM, *n* = 3. ***p < 0.001 *vs.* MCS group. ^#^*p* < 0.05, ^##^*p* < 0.01, ^###^*p* < 0.001 vs. MCD group. MCS: methionine and choline supplemented; MCD: methionine and choline-deficient.

To investigate the beneficial effects of esculin on fibrosis in mice with MCD diet-induced NASH, Masson trichrome and immunochemistry staining were performed. Based on Masson trichrome staining, esculin reduced the expression of collagen fibres in the liver tissue of mice that were administered the MCD diet (Figure 3(A)). Further, immunohistochemistry revealed that the levels of fibronectin and collagen 4A1 were increased in the liver tissue of the MCD diet-fed mice; these changes were markedly inhibited by esculin treatment (Figure 3(B,C)). Subsequently, the effect of esculin on inflammation in mice with MCD diet-induced NASH was assessed. Based on immunochemistry staining, positive cells marked with F4/80 were significantly increased in the MCD group, while inflammatory cell infiltration was decreased in esculin-treated mice (Figure 3(D)). Consistent with the above results, esculin attenuated the increase in the levels of TNF-α, IL-6, and IL-1β in the liver tissue of mice fed the MCD diet (Figure 3(E–G)). Such findings indicate that fibrosis and inflammation induced by the MCD diet were ameliorated by esculin.

### Esculin inhibits the acetylation of NF-κB in MCD diet-fed mice

**Figure 4.**
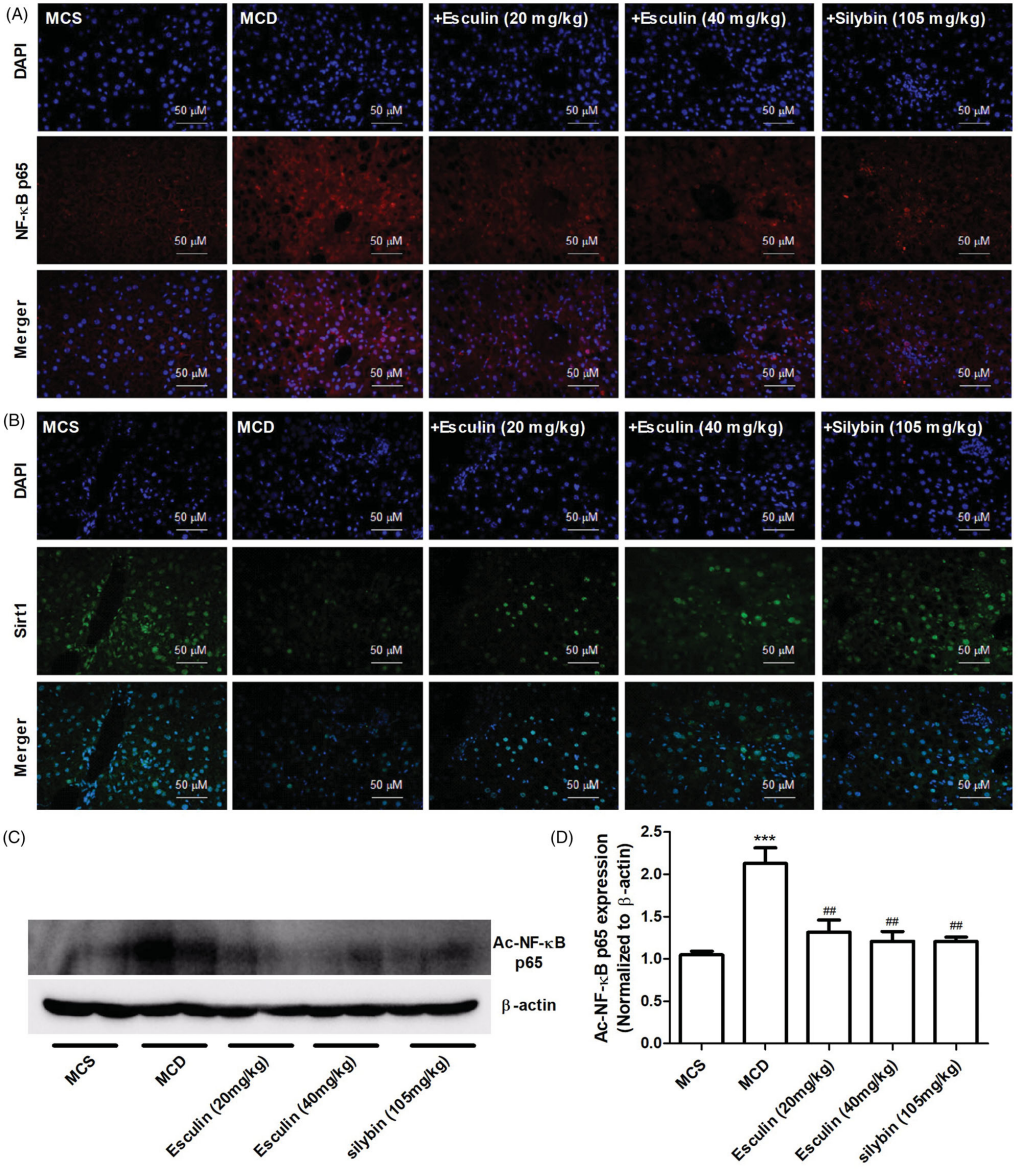
Esculin inhibits the acetylation of NF-κB in MCD diet-induced mice. (A) Representative immunofluorescence images of the NF-κB p65 protein in the liver of each group (400× magnification, scale bar: 50 µm). (B) Representative immunofluorescence images of the Sirt1 protein in the liver of each group (400× magnification, scale bar: 50 µm). (C) Western Blot analysis of ac-NF-κB p65. (D) Densitometric analyses of ac-NF-κB p65. Data are expressed as mean ± SEM, *n* = 3. ***p < 0.001 *vs.* MCS group. ^##^*p* < 0.01 *vs.* MCD group. MCS: methionine and choline supplemented; MCD: methionine and choline-deficient.

Because Sirt1-mediated acetylation of NF-κB (ac-NF-κB) has an important role in the activation of inflammatory signals, we detected the expression of Sirt1, NF-κB, and ac-NF-κB in the liver tissue. As shown in Figure 4(A,B), the levels of NF-κB expression and its nuclear translocation were increased, and the level of Sirt1 expression was decreased in MCD diet-fed mice. However, these effects were found to be markedly inhibited by esculin treatment. Based on the western blot results, the administration of esculin markedly inhibited the upregulated protein expression levels of ac-NF-κB in the MCD diet-fed mice (Figure 4(C,D)). These data indicate that esculin regulates the Sirt1/ac-NF-κB signalling pathway.

### Esculin improves FFA-induced hepatocyte inflammation

**Figure 5.**
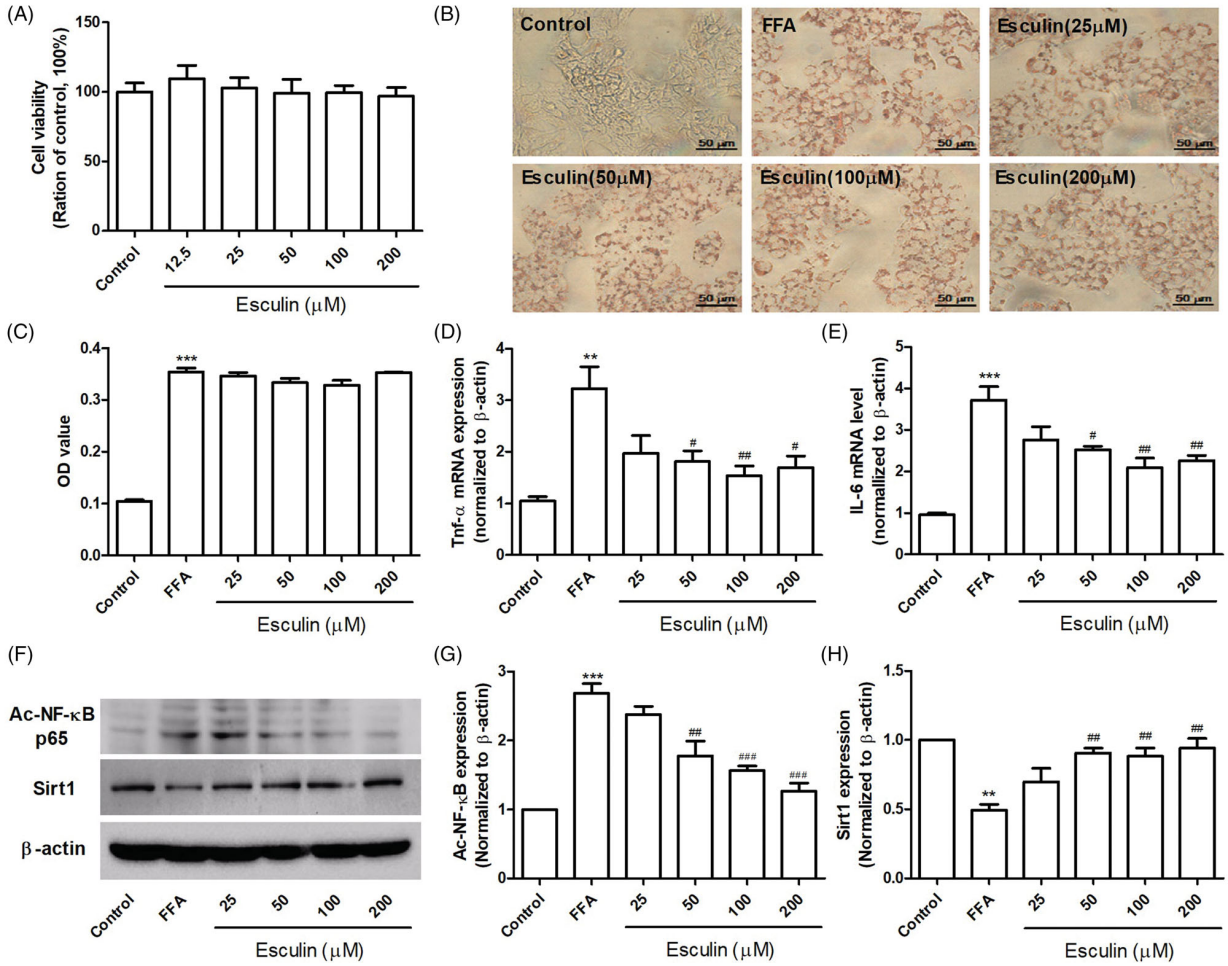
Esculin improves FFA-induced hepatocyte inflammation. (A) Effect of various concentrations of esculin on cell viability. (B) Representative Oil Red O staining images (400× magnification, scale bar: 50 µm). (C) Quantitative analysis of Oil Red O staining. (D) The mRNA levels of TNF-α. (E) The mRNA levels of IL-6. (F) Western Blot analysis of ac-NF-κB and Sirt1. (G) Densitometric analyses of ac-NF-κB. (H) Densitometric analyses of Sirt1. Data are expressed as mean ± SEM, *n* = 3. **p < 0.01, ***p < 0.001 *vs.* control group. ^#^*p* < 0.05, ^##^*p* < 0.01, ^###^*p* < 0.001 *vs.* FFA group. FFA: free fatty acid.

Whether esculin inhibits lipid synthesis, inflammation, and fibrosis induced by FFA *in vitro* remains unclear. MTT analysis revealed that esculin (12.5–200 µM) had no effect on the viability of HepG2 cells (Figure 5(A)). Further, based on Oil Red O staining, HepG2 cells incubated with FFA (500 µM) had increased lipid accumulation; however, esculin treatment (12.5–200 µM) had no effect on lipid accumulation (Figure 5(B,C)). RT-qPCR revealed that treating HepG2 cells with esculin (50, 100, and 200 µM) significantly restored the increase in TNF-α and IL-6 expression (Figure 5(D,E)). Similarly, we confirmed that the expression of ac-NF-κB was markedly upregulated while that of Sirt1 was downregulated in the FFA-treated HepG2 cells. However, these changes were found to be ameliorated in esculin-treated cells (Figure 5(F–H)).

### Esculin improves FFA-induced hepatocyte inflammation and fibrosis via Sirt1

**Figure 6.**
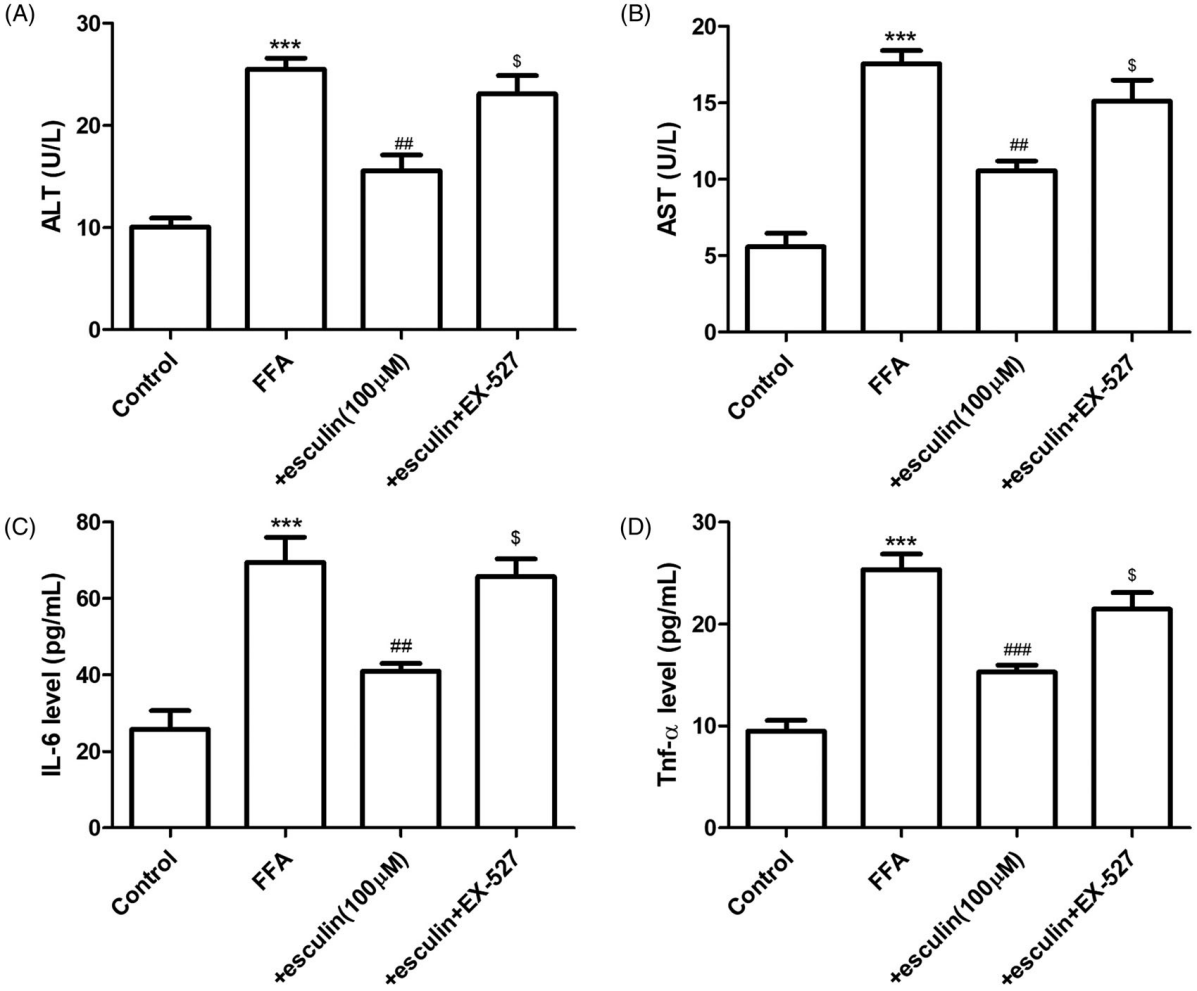
Sirt1 inhibitor abolishes the protective effect of esculin against FFA-induced hepatocyte inflammation. (A) Level of ALT in the cell culture supernatant. (B) Level of AST in the cell culture supernatant. (C) Level of IL-6 in the cell culture supernatant. (D) Level of TNF-α in the cell culture supernatant. Data are expressed as mean ± SEM, *n* = 3. ***p < 0.001 *vs.* control group. ^##^*p* < 0.01, ^###^*p* < 0.001 *vs.* FFA group. ^$^*p* < 0.05 *vs.* + esculin group. FFA: free fatty acid.

**Figure 7.**
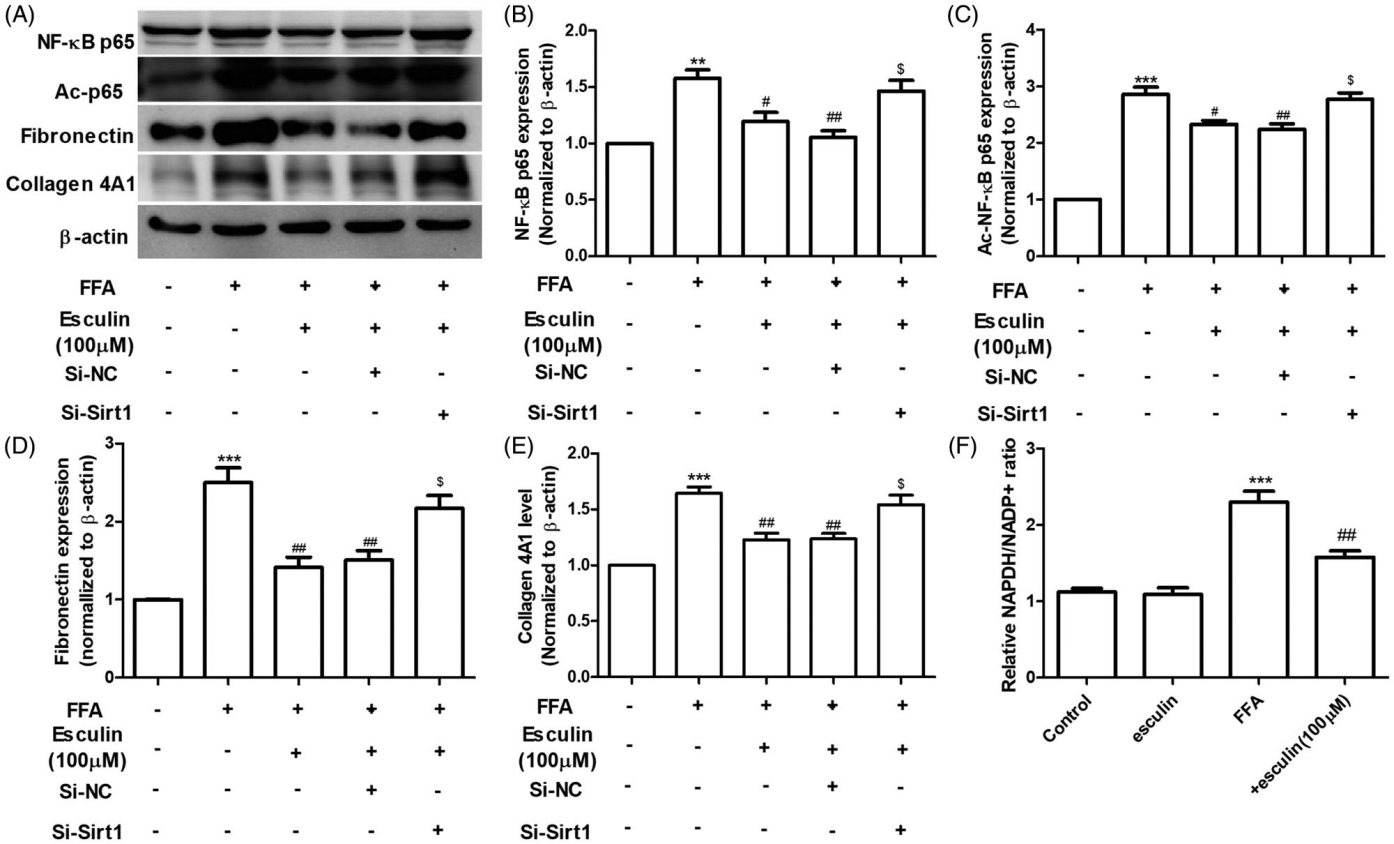
Sirt1 silencing affects the activity of esculin on FFA-induced inflammation and fibrosis. (A) Western blot analysis of NF-κB p65, ac-NF-κB p65, fibronectin, collagen 4A1, and β-actin. (B) Densitometric analyses of NF-κB p65. (C) Densitometric analyses of ac-NF-κB p65. (D) Densitometric analyses of fibronectin. (E) Densitometric analyses of collagen 4A1. (F) Relative NADPH/NADP^+^ ratio. Data are expressed as mean ± SEM, *n* = 3. **p < 0.01, ***p < 0.001 *vs.* control group. ^#^*p* < 0.05, ^##^*p* < 0.01 *vs.* FFA group. ^$^*p* < 0.05 *vs.* FFA + esculin + Si-NC group. FFA: free fatty acid, NC: negative control.

We proceeded to investigate the role of Sirt1 in esculin-mediated inhibition of FFA-induced inflammation. Briefly, HepG2 cells were incubated with EX-527 (10 µM), a selective inhibitor of Sirt1, for 6 h followed by FFA (500 µM) in the presence and absence of esculin (100 µM). As shown in Figure 6(A–D), the esculin-induced downregulation of AST, ALT, IL-6, and TNF-α in the cell culture supernatant was reversed by EX-527. To further reveal the underlying molecular mechanism of the protective effect of esculin on FFA-induced inflammation and fibrosis, Sirt1 expression in HepG2 cells was silenced by siRNA. As shown in Figure 7(A–E), esculin treatment inhibited the increase in the levels of NF-κB, ac-NF-κB, fibronectin, and collagen 4A1 expression in HepG2 cells exposed to FFA (500 µM). Further, esculin lost its protective effects when Sirt1 was silenced in HepG2 cells. Because the activity and expression level of Sirt1 were regulated by NAD ^+^ content (Revollo and Li 2013), the effect of esculin (100 µM) on the NADPH/NADP ^+^ ratio was measured. The increase in NADPH/NADP ^+^ ratio was significantly suppressed after HepG2 cells were treated with esculin (Figure 7(F)). Such findings indicate that esculin exerts its anti-inflammatory and anti-fibrotic effects by restoring Sirt1.

## Discussion

NAFLD is the hepatic manifestation of metabolic syndrome and is often accompanied by hyperlipidaemia, insulin resistance, hyperlipidaemia, and obesity (Romero et al. 2020). NASH may progress to cirrhosis and hepatocellular carcinoma, which lead to liver-related death (Ipsen et al. 2020). Many drugs administered to treat hyperlipidaemia and diabetes have a certain therapeutic effect on NASH (Kumar et al. 2020; Ozutsumi et al. 2020). Currently, some anti-NASH drugs are in phase 2 and phase 3 clinical trials. However, the effects of each drug on the histological improvement of NASH, including lipid accumulation, inflammation, and liver fibrosis, have not been satisfactory (Noureddin et al. 2020). Excessive formation of lipid droplets in liver cells is associated with increased lipid droplet synthesis, decreased lipid droplet metabolism and beta-oxidation of fatty-acid, or impaired TG secretion (Rong et al. 2015). FFA plays a major role in lipid droplet synthesis. Thus, reducing FFA synthesis or increasing the beta-oxidation of FFA may be a potential strategy for treating NASH. In this study, we demonstrated that esculin not only reduced the MCD diet-induced lipid accumulation in the liver, but also inhibited the levels of FASN, CD36, and ADRP expression in mice fed an MCD diet. These results suggest that esculin alleviated the MCD diet-induced lipid accumulation in the liver. Although the *in vitro* experiment demonstrated that esculin had no effect on FFA-induced lipid accumulation, it was found to have a significant protective effect against the expression of inflammatory factors and fibrotic factors.

NF-κB is a homo- or heterodimeric complex formed by the Rel-like domain-containing proteins, RelA (p65), NF-κB1 (p50), NF-κB2 (p52), c-Rel, and RelB (Mitchell et al. 2016). Previously, NF-κB was reported to participate in the regulation of the expression of a wide range of genes involved in immune responses, inflammation, and fibrosis (Wu et al. 2019). The activation of NF-κB is regulated by multiple pathways. Under normal circumstances, NF-κB and its inhibitory protein, IκB, combine to form an inactivation complex. Under stress, IκB is phosphorylated and degraded, and free NF-κB enters the cell nucleus to perform transcriptional functions (Jimi et al. 2019). The transcription activity of NF-κB is regulated by many post-translational modifications, such as methylation, acetylation, phosphorylation, and ubiquitination (Tafani et al. 2013). Previous research proved that acetylation at lysine 310 is required for the full transcriptional activity of p65, without affecting DNA binding (Huang et al. 2010).

In the present study, we found that a MCD diet or FFA increased the level of acetylated NF-κB at lysine 310 and inflammatory factor expression *in vivo* and *in vitro*. Treatment with esculin was found to ameliorate the increased ac-NF-κB and inflammatory factor levels. Sirt1 is known to play a major role in regulating ac-NF-κB and the activation of an inflammatory signal (Jiang et al. 2020). Therefore, the level of Sirt1 expression was examined. Esculin was found to restore the MCD diet- or FFA-induced downregulation of Sirt1 *in vitro* and *in vivo*. Furthermore, this protective effect of esculin against FFA-induced inflammation and fibrosis was abrogated when the Sirt1 in HepG2 cells was silenced. These data suggest that esculin inhibits FFA-induced inflammation and fibrosis in HepG2 cells by regulating Sirt1.

Although we demonstrated that esculin reduces the acetylation level of NF-κB p65 *in vivo* and *in vitro*, and proved that interfering with the expression of Sirt1 cancels the regulatory effect of esculin on the acetylation level of NF-κB p65, several experiments, such as molecule-protein interaction and molecular docking experiments, must be carried out to explore how esculin regulates the deacetylation of NF-κB p65 via Sirt1. Currently, complete research on the toxicological effects of esculin has not been performed. Based on a report, Balb/C mice administered esculin (20 or 40 mg/kg) displayed no significant genotoxicity in liver and kidney cells (Mokdad Bzeouich et al. 2016). Such findings indicate that esculin does not exhibit cytotoxic effects; however, its toxicology must be further explored.

## Conclusions

Based on the findings of the present study, esculin protects against MCD diet-induced NASH by regulating Sirt1/ac-NF-κB p65 signalling. Such findings indicate that esculin could be employed as a therapeutic agent for preventing and treating NASH. However, further studies are needed to evaluate its effect in patients with NASH.
